# Efficacy of Silexan in Patients with a Major Depressive Episode – First Results from a Multi-centre, Double-blind, Randomised, Placebo- and Reference-controlled Phase III Trial

**DOI:** 10.1192/j.eurpsy.2024.479

**Published:** 2024-08-27

**Authors:** S. Kasper, E. Seifritz, H.-P. Volz

**Affiliations:** ^1^Molecular Neuroscience, Medical University of Vienna, Vienna, Austria; ^2^Psychiatry, University of Zürich, Zürich, Switzerland; ^3^Department of Psychiatry, Psychotherapy and Psychosomatic Medicine, HospitaL OF PSYCHIATRY, Werneck, Germany

## Abstract

**Introduction:**

Silexan [1], an essential oil from *Lavandula angustifolia* flowers, is the active substance of a medicinal product for oral use in the treatment of anxiety disorders. It has been shown to be effective in the treatment of patients suffering from mixed anxiety and depression.

[1] Silexan® is a special essential oil from Lavandula angustifolia, Dr. Willmar Schwabe GmbH & Co. KG, Karlsruhe, Germany

**Objectives:**

The trial (ISRCTN36202964) was conducted to investigate the antidepressant efficacy of Silexan in patients with a major depressive episode compared to placebo and Sertraline.

**Methods:**

Adult patients (≥18 years) suffering from a major depressive episode of mild to moderate severity according to ICD‑10 were included. Further inclusion criterion was a total score of 19 – 34 points in the Montgomery-Asberg-Depression Rating Scale (MADRS). Randomised patients took 80 mg Silexan, 50 mg Sertraline, or placebo once daily over 8 weeks. Primary efficacy endpoint was the change of the MADRS total score between baseline and week 8. Response (a reduction of the MADRS total score ≥50%), remission (MADRS total score <10 at the end of the treatment), the Patient Health Questionnaire PHQ-9, the Beck Depression Inventory, the Clinical Global Impressions, and the Sheehan Disability scale served as secondary endpoints.

**Results:**

The full analysis set consisted of 498 patients. Between the start and end of treatment, the MADRS total score decreased by 12.1 (13.3, 11.0) points (adjusted mean, 95% confidence interval) in patients treated with Silexan, by 12.6 (13.7, 11.5) points in patients treated with Sertraline, and by 9.95 (11.1, 8.77) points under placebo. The confirmatory analysis proved that Silexan was significantly superior to placebo (p<0.01, ANCOVA). Internal validity could be shown since the treatment effects of the active comparator Sertraline were also more pronounced compared to placebo (p<0.01). There were no relevant differences between Silexan and Sertraline. Response was achieved by 53.5% of the patients in the Silexan group, by 54.0% of the patients in the Sertraline group, and by 41.5% of the patients in the placebo group. 44.4% of the patients treated with Silexan were remitter, compared to 45.2% under Sertraline and 32.6% under placebo. In both active treatment groups responder and remission rates were higher than in the placebo group (p < 0.05). Results of the secondary endpoints were in line with the results of the primary endpoint.

**Conclusions:**

In a large phase III clinical trial, Silexan was more effective than placebo and not different to Sertraline in patients with a major depressive episode. Treatment effects were clinically relevant.

**Disclosure of Interest:**

S. Kasper Consultant of: In the past 3 years Dr Kasper served as a consultant or on advisory boards for Angelini, Biogen, Boehringer, Esai, Janssen, IQVIA, Mylan, Recordati, Rovi, Sage and Schwabe; and he has served on speakers bureaus for Angelini, Aspen Farmaceutica S.A., Biogen, Janssen, Recordati, Schwabe, Servier, Sothema, and Sun Pharma., Speakers bureau of: In the past 3 years Dr Kasper served as a consultant or on advisory boards for Angelini, Biogen, Boehringer, Esai, Janssen, IQVIA, Mylan, Recordati, Rovi, Sage and Schwabe; and he has served on speakers bureaus for Angelini, Aspen Farmaceutica S.A., Biogen, Janssen, Recordati, Schwabe, Servier, Sothema, and Sun Pharma., E. Seifritz Consultant of: Schwabe, Janssen, Speakers bureau of: Schwabe, Janssen, H.-P. Volz Consultant of: Schwabe, Janssen, Speakers bureau of: Schwabe, Janssen

